# In vitro and in silico assessment of the effect of *WWOX* expression on invasiveness pathways associated with AP-2 transcription factors in bladder cancer

**DOI:** 10.1186/s12894-021-00806-7

**Published:** 2021-03-10

**Authors:** Żaneta Kałuzińska, Damian Kołat, Katarzyna Kośla, Magdalena Orzechowska, Andrzej K. Bednarek, Elżbieta Płuciennik

**Affiliations:** Department of Molecular Carcinogenesis, Medical University of Lodz, 90-752 Lodz, Poland

**Keywords:** Bladder cancer, WWOX, TFAP2A, TFAP2C, AP-2alpha, AP-2gamma, AP-2 transcription factors

## Abstract

**Background:**

WW Domain Containing Oxidoreductase (*WWOX*) belongs to the unusual tumor suppressors, whose molecular function is not fully understood in bladder cancer, especially regarding interaction with Activator Protein 2 (AP-2) α/γ transcription factors. Thus, using lentiviral systems we created an in vitro model overexpressing or downregulating *WWOX* in CAL-29 cell line to assess invasiveness pathways. Surprisingly, while *WWOX* overexpression was accompanied with increased expression of both AP-2 factors, its downregulation only affected AP-2α level but not AP-2γ which remained high.

**Methods:**

Using cellular models and unpaired t-test or Wilcoxon test, we investigated significant changes in biological processes: clonogenicity, extracellular matrix adhesion, metalloproteinases activity, 3D culture growth, proliferation, mitochondrial redox potential and invasiveness. Relative gene expression acquired through Real-Time qPCR has been analyzed by Welch's t-test. Additionally, using oncoprint analysis we distinguished groups for bioinformatics analyzes in order to perform a follow-up of in vitro experiments.

**Results:**

Downregulation of *WWOX* in bladder cancer cell line intensified ability of single cell to grow into colony, mitochondrial redox potential and proliferation rate. Moreover, these cells shown elevated pro-MMP-2/9 activity but reduced adhesion to collagen I or laminin I, as well as distinct 3D culture growth. Through global in silico profiling we determined that WWOX alters disease-free survival of bladder cancer patients and modulates vital processes through AP-2 downstream effectors.

**Conclusions:**

Our research indicates that *WWOX* possesses tumor suppressor properties in bladder cancer but consecutive examination is required to entirely understand the contribution of AP-2γ or AP-2α.

**Supplementary Information:**

The online version contains supplementary material available at 10.1186/s12894-021-00806-7.

## Background

The incidence of bladder cancer (BLCA) has been decreasing over recent years in the US yet in some European countries it is steadily rising [1]. Nevertheless, it remains the sixth most common cancer among men [1]. According to the American Cancer Society, almost 81,000 new cases of bladder cancer were diagnosed in 2018, of which 17,000 were fatal [2].

Several subtypes of BLCA are recognized, and these are distinguished on the basis of their molecular profile. The University of North Carolina (UNC) classification assumes two subtypes (luminal and basal bladder cancer), while the MD Anderson Cancer Center (MDA) recognizes three (basal, luminal and TP53-like) and The Cancer Genome Atlas (TCGA) database four [3]. BLCA is a heterogeneous disease in terms of its molecular and clinicopathological status; it is associated with disruptions of pathways, with the best known being FGFR3/RAS, PI3K/AKT/mTOR or TP53/RB1 [4].

One of many tumor suppressor genes whose function remains unclear in bladder cancer (especially at the molecular level) is *WWOX*. The gene is located in a common fragile site FRA16D (16q23.1-q23.2) and belongs to the group of tumor suppressor genes inactivated through haploinsufficiency. Mutations inactivating both *WWOX* alleles have not been found in cancer; however, the loss of one allele is sufficient to affect its normal biological function [5]. One of the mechanisms regulating *WWOX* expression in bladder cancer is methylation of both the promoter region and the first exon of the gene [6, 7]. In addition, our previous research proved a frequent (47.5%) loss of heterozygosity (LOH) located in intron 1 (microsatellite marker D16S518) of the *WWOX* gene, together with positive correlation between the levels of *WWOX* and *CCND1* mRNA in bladder cancer patients [7]. Furthermore, in vitro study revealed that cigarette smoke extract (CSE) treatment could inhibit the expression of *WWOX* in a time-dependent manner, through the induction of CpG islands methylation near to the transcription start site (TSS) [8]. Until now, only one publication reported that WWOX may regulate cell cycle and apoptosis but also reduces tumorigenicity in mice; research was done on 5637 bladder cancer cell line (grade 2) [9]. Studies on bladder cancer patients indicate a relationship between progressive loss of WWOX protein expression and higher tumor grade, more advanced stage, larger tumor size and shorter progression-free survival [10].

WWOX is known as a global modulator of gene expression and cell metabolism. It encodes a protein containing two N-terminal WW domains and a centrally-located short chain dehydrogenase domain (SDR), suggesting that it acts as a steroid dehydrogenase and participate in the regulation of steroid hormone receptor interactions [11–13]. The first WW domain interacts with a number of partner proteins, including ERBB4 [14, 15], transcription factor AP-2γ [16, 17], YAP [14], c-Jun [18], HIF1α [19, 20], p73 [20, 21], DVL2 [22], RUNX2 [23], SMAD3 [24] and GLI [25].

A recent study, by Abu-Odeh et al. identified a set of 240 proteins that may be able to bind with WWOX, either classically (via PPxY or LPxY motifs) or through non-canonical proline-rich motifs. These new partners are believed to be involved in the transcription process, RNA processing and splicing, chromatin remodeling, metabolism, and signaling pathways [20].

One of WWOX partners is AP-2γ which belongs to the AP-2 transcription factor family, a crucial group of TFs that regulate gene expression during early development but also in carcinogenesis. Another member of the AP-2 family, commonly interacting with WWOX, is AP-2α. Of the two, AP-2γ has been described in more detail, which is presumably due to greater PPxY motif affinity demonstrated by this protein (^56^PPPYFPPPY^64^) in contrast to proline-rich motif found in AP-2α (^59^PPPY^62^) [16]. Their transcriptional transactivation function is suppressed by the interaction with WWOX through their sequestration in the cytoplasm [16]. Both of the AP-2 factors possess ambiguous functions [26]. Despite the discrepancies observed in the literature, the prevailing opinion is that AP-2α is more likely to be downregulated during tumor progression [27, 28]; however, AP-2γ is believed to be upregulated during progression (but not initiation [16]), suggesting that it has an oncogenic character. This pattern was confirmed in breast carcinoma, where AP-2γ nuclear translocation resulting from WWOX inactivation was observed during tumor progression [16].

The aim of this study was to determine the impact of WWOX on invasiveness pathways in the grade 4 bladder cancer cell line CAL-29. To provide clearer results, two in vitro models were developed: one with low *WWOX* expression and the other with overexpression. The differential *WWOX* gene expression was found to be associated with various levels of *TFAP2A* (encoding AP-2α) and *TFAP2C* (encoding AP-2γ) expression. Therefore, an in silico analysis was also performed on clinical data obtained from TCGA database since the tumor profile is changing globally in terms of *WWOX* and *TFAP2C* levels which corresponded to our research model.

## Methods

### Cell culture

The CAL-29 cell line was purchased from the DSMZ (Deutsche Sammlung von Mikroorganismen und Zellkulturen). This urinary bladder cancer cell line was established from transitional cell carcinoma, luminal molecular subtype (histological grade 4, stage pT2). Cells were grown on DMEM medium supplemented with 10% heat-inactivated FBS (Fetal Bovine Serum), 1% PSN (Penicillin 50 μg/mL; Streptomycin 50 μg/mL; Neomycin 100 μg/mL). Cells were incubated at 37 °C in a humidified atmosphere of 5% CO_2_.

### Acquisition of BLCA patients’ data

Expression (RNAseqV2, RSEM normalized, data status of 28th Jan 2016) and clinical data of 408 BLCA patients were obtained from TCGA repository (https://gdac.broadinstitute.org/). Detailed characteristics of BLCA patients are shown in Additional file 1.

### Survival analysis

Disease-free survival (DFS) analysis was performed regarding the significance of *WWOX*, *TFAP2A* and *TFAP2C* expression alterations combined together. BLCA patients were divided into eight subgroups according to the expression level of *WWOX*, *TFAP2A* and *TFAP2C* determined through oncoprint analysis at cBioPortal based on mRNA expression z-scores (RNAseq V2, RSEM normalized, z-score threshold ± 0) [29, 30]. Table 1 shows detailed specification of groups with expression levels of each gene and number of patients assigned.

**Table 1:** Subgroup specification of BLCA patients with *WWOX*, *TFAP2A* and *TFAP2C* expression levels

Group	Number of patients	*WWOX*	*TFAP2A*	*TFAP2C*
A	58	Low	High	High
B	84	Low	Low	Low
C	37	High	High	High
D	38	High	Low	Low
E	24	High	High	Low
F	47	High	Low	High
G	39	Low	High	Low
H	81	Low	Low	High

The survival curves for the groups were fitted with survfit() (survival R package) using days to last follow-up as time variable, person neoplasm cancer status as event indicator (dummy coding) and group assignment as strata. Kaplan–Meier plots presenting the expected duration of time until recurrence of a disease for all groups together and chosen pairwise comparisons were generated via survminer R package. Significance of DFS between specific pairs of survival curves was tested with log-rank test. Hazard ratios (HR) with 95% confidence intervals (95% CI) and *p* values were computed using univariate Cox proportional hazards model accompanied by test of proportional hazards with Schoenfeld residuals (coxph() and coxzph() functions).

### AP-2α/AP-2γ downstream targets analysis: clustering of differentially expressed genes

To determine targets for both transcription factors, we combined three databases: Gene Transcription Regulation Database (GTRD, version 19.10 [31, 32]]), TRANScription FACtor database (TRANSFAC, version 2019.2) and Transcriptional Regulatory Relationships Unraveled by Sentence-based Text mining (TRRUST, version v2). Excluding duplicates, there were 5175 and 4810 targets for AP-2γ and AP-2α, respectively.

Differences in expression of AP-2γ and AP-2α downstream targets were determined using ExpressCluster software (http://cbdm.hms.harvard.edu/). The analysis was performed separately for each transcription factor targets. Primary input was filtered with minimum of three-fold-change in expression followed by applying K-means clustering algorithm with z-norm signal transformation method, rank correlation as distance metric, 1000 iterations and clusters (K) = 18. Finally, for the most interesting clusters the gene ontology analysis in terms of biological processes was performed using the Protein ANalysis THrough Evolutionary Relationships (PANTHER) Classification System (Annotation Data Set: GO-Slim Biological Process) [33].

### Transduction

The overexpressed *WWOX* profile was obtained using the GIPZ Lentiviral™ system (pLenti-GIII-CMV-GFP-2A-Puro) and Puro-Blank Lentivirus used as a control (Applied Biological Materials Inc.). Transduction was performed in starving medium containing 8 µg/mL polybrene and lentiviral particles at MOI = 3. After 24-h transduction, the medium was changed for full medium. After 72-h, antibiotic-based clone selection (puromycin, 1 µg/mL) was performed.

For downregulated *WWOX* profile, sgRNA CRISPR/Cas9 (pLenti-U6-sgRNA-SFFV-Cas9-2A-Puro) was used, with Scrambled sgRNA CRISPR/Cas9 (Applied Biological Materials Inc.) as control. Transduction was performed in starving medium containing 8 µg/mL polybrene and lentiviral particles at MOI = 2. Transduction medium was changed into full medium after 24-h of incubation. This was followed by antibiotic-based clone selection (puromycin, 1 µg/mL) after 72-h.

### Protein extraction and western blotting

Cells were lysed using RIPA buffer with protease and phosphatase inhibitor cocktail and phenylmethylsulfonyl fluoride (PMSF). Protein concentration was determined with the Bradford method (Bio-Rad Laboratories). SDS-PAGE was followed by semi-dry transfer (45 min, 250 mA, Fast Blot, Biometra) of the proteins to a PVDF membrane (Sigma-Aldrich). The process was supported by Whatman® Lens Cleaning Tissue (Sigma Aldrich), the use of which requires pre-soaking in TB 10 × transfer buffer. Protein transfer was confirmed by transient staining with Ponceau red. Precluding of non-specific binding of the antibodies was accomplished via membrane blocking with non-fat milk (5% solution) in TBST buffer 1 × .

Anti-WWOX/AP-2γ/AP-2α (Thermo-Fisher; catalogue numbers: PA5-29701, PA5-49862 and MA5-14856, respectively) were added as primary antibodies at a dilution of 1:1000 to 1% non-fat milk in TBST buffer solution. Overnight incubation was followed by three times washing using TBST buffer. Following this, the PVDF membranes were incubated with secondary anti-rabbit antibodies conjugated with alkaline phosphatase (1:30,000) and the analyzed protein were visualized with Novex® AP Chromogenic Substrate (Invitrogen).

Anti-GAPDH antibody was used as reference (sc-59540, Santa Cruz Biotechnology Inc.). The relative amount of protein was determined by densitometric analysis with ImageJ software.

### RNA isolation and cDNA synthesis

RNA isolation was performed using TRIzol® reagent (Invitrogen). The ImProm-II™ Reverse Transcription System (Promega) was used for cDNA synthesis. The samples (10 µg of total RNA) were mixed with 6 µL Random Hexamers and 5 µL oligo(dT)_15_ (both at concentration 0.05 µg/µL) and incubated at 70 °C for 10 min. Following this, 1 × buffer M-MLV, dNTPs, and reverse transcriptase ImProm RT-II™ were added according to the manufacturer's protocol. cDNA synthesis was performed at the following conditions: annealing at 25 °C for five minutes, elongation of the products at 42 °C for 60 min, completion at 70 °C for 15 min. 50 μL of water was added to each sample and the samples were stored at − 20 °C.

### Real-time qPCR

The expression levels of the target and reference genes were determined using GoTaq® qPCR Master Mix (Promega). The expression levels of seven genes (*ACVR1B*, *CRABP2*, *CDKN1A*, *TP63*, *AP1M2*, *IKBKB*, *SMAD4*) were evaluated, relative to three reference genes (*H3F3A*, *RPLP0*, *RPS17*). Universal Human Reference RNA (Stratagene, La Jolla, CA, USA) was used as a calibrator. Based on literature data and bioinformatic databases (e.g. TRRUST, GeneCards), a group of target genes regulated by transcription factor AP-2γ was selected: *p21/WAF1/CDKN1A*, *TP63*, *SMAD4*, *CRABP2*, *ACVR1B*, *AP1M2*, *IKBKB*. The relative expression level of genes was calculated by Relative Expression Software Tool Multiple Condition Solver (REST-MCS) (http://rest.gene-quantification.info/). Primer sequences, PCR conditions and product length are presented in Tables 2 and 3.

**Table 2:** Real-time qPCR primer sequences, amplification conditions and product length for reference genes

Gene	Primer sequence	Product [bp]	Annealing [°C]	Reading [°C]
*H3F3A*	^5′^ACGGATTACACCTTCCCACTTGCTAAAAGGTC^3′^ ^5′^AGCCACAAAGGCAGATGGATCAGCCAAG^3′^	69	65	72
*RPLP0*	^5′^AGGACTTTAAAAGATCTGCGCTTCCAGAG^3′^ ^5′^ACCAGATAGGCCTCACTTGCCTCCTGC^3′^	76	65	72
*RPS17*	^5′^AAGCGCGTGTGCGAGGAGATCG^3′^ ^5′^TCGCTTCATCAGATGCGTGACATAACCTG^3′^	87	64	72

**Table 3:** Real-time qPCR primer sequences, amplification conditions and product length for target genes

Gene	Primer sequence	Product [bp]	Annealing [°C]	Reading [°C]
*ACVR1B*	^5′^CTGACCCTTCCATTGAGGAAAT^3′^ ^5′^CCGCAGTGCCTCATAACTC^3′^	95	60	60
*CRABP2*	^5′^CCTGTAAGAGCCTGGTGAAA^3′^ ^5′^ATCGTTGGTCAGTTCTCTGG^3′^	110	60	60
*CDKN1A*	^5′^GACAGACAACTCACTCGTCAAA^3′^ ^5′^CATGGCGCCTGAACAGAA^3′^	86	60	60
*TP63*	^5′^GAAACCAGAGATGGGCAAGT^3′^ ^5′^ATGCTATCTTCATCCGCCTTC^3′^	95	60	60
*AP1M2*	^5′^AAGCCACTGATCTGGATTGAG^3′^ ^5′^CCTTGGCCTTGACCATGA^3′^	76	60	60
*IKBKB*	^5′^CTGTATCCTTCAAGAGCCCAAG^3′^ ^5′^CCGGTTGCAATCTTCCTTCA^3′^	106	60	60
*SMAD4*	^5′^ATGGATACGTGGACCCTTCT^3′^ ^5′^ATGTGCAACCTTGCTCTCTC^3′^	97	60	60

### Clonogenic assay

All variants of the CAL-29 cell line were seeded (5 × 10^2^/well) onto 6-well plates in full culture medium and incubated (10 days, 37 °C, 5% CO_2_). The cells were fed with full medium every four days. The cells were then fixed with 4% paraformaldehyde in PBS solution. Staining was conducted with 0.005% crystal violet (15 min, RT) and cell colonies were counted using ImageJ software. The assay was performed in triplicate for each cell variant as described previously [34].

### Cell adhesion assay

The adhesiveness of CAL-29 cells (with overexpressed or downregulated *WWOX* gene) to four extracellular matrix proteins: collagen I, collagen IV, laminin I and fibronectin was evaluated using Corning® BioCoat™ plates. The cells were seeded (3 × 10^5^/well) onto 24-well adhesion plate and allowed to adhere via incubation (4 h, 37 °C, 5% CO_2_). Adherent cells were stained (0.1% crystal violet, 10 min) and their cellular adhesion was measured spectrophotometrically at 570 nm (BioTek) after extraction in 10% acetic acid.

### Zymography

Cells were seeded in 6-well plates (1.5 × 10^6^/well) and cultured for 80% confluency. Medium was changed into starving medium and the cells were cultured for 48 h (7 °C, 5% CO_2_). The medium was then collected to measure protein concentration using Qubit Protein Assay on the Qubit 2.0 Fluorometer (Life Technologies).

A gelatin (2 mg/mL) was embedded in the resolving gel during preparation of the acrylamide gel (10%). Briefly, 8 µg of protein was added to each lane and separated during electrophoresis, then SDS was removed from the gel by incubation in 2.5% Triton X-100, followed by incubation in buffer (0.5 M Tris–HCl, 2 M NaCl, 50 mM CaCl_2_, pH 7.5), overnight at 37 °C. The resulting zymogram was subsequently stained with Coomassie Brilliant Blue R-250, the gel was then washed with bleaching solution (methanol:acetic acid:water, 3:1:6). The gel areas where gelatin was enzymatically degraded appeared as clear bands against a stained dark blue background. A protein marker was used to measure the molecular weights of the MMPs; their activity was measured using ImageJ software. The assay was performed in duplicate for each cell variant.

### 3D culture assay

To estimate spatial growth potential, 15 × 10^3^ cells with different *WWOX* levels were seeded on 96-well plate on a thick (2 mm) solidified layer of ECM matrix (Geltrex, Gibco) and incubated for 14 days. Cells were fed every four days by 2% solution of ECM matrix. After this period, the cells were observed under the microscope and photographed. The assay was performed in triplicate for each cell variant.

### Proliferation and cell viability assays with an evaluation of mitochondrial redox activity

Proliferation rate was evaluated using 5-bromo-2′-deoxyuridine (BrdU). This assay was performed on fixed cells, incubated (one hour, 37 °C, 5% CO_2_) with europium labelled anti-BrdU antibody and visualized at wavelength OD 340 nm using VICTOR™ X4 Multilabel Plate Reader (Perkin Elmer). The mitochondrial redox activity was estimated with Presto reagent (Invitrogen). Cell viability was measured spectrophotometrically at a wavelength of 570 nm every 10 min on a VICTOR™ X4 Multilabel Plate Reader (Perkin Elmer).

### Invasion assay

The Corning® BioCoat™ Matrigel® Invasion Chambers with 8.0 μm PET membrane was used to assess the role of the *WWOX* gene in CAL-29 invasiveness. Cells were suspended in starving medium and then seeded (2 × 10^5^/well) onto the inner compartment of each insert, after adding full culture medium to the wells. The plate was incubated (48 h, 37 °C, 5% CO_2_). Following this, the cells attached to the outer side of the membrane were stained with 0.1% crystal violet. The inserts were transferred to 200µL of extraction solution (10% acetic acid) and incubated on an orbital shaker. The intensity of stain extract from the cells was measured spectrophotometrically (OD 560 nm, BioTek). The assay was performed in triplicate for each cell variant.

### Statistical analysis

Levene's test was used to test the homogeneity of variance. The normality of distribution was determined by the Shapiro–Wilk test; depending on the result, either the unpaired t-test or Wilcoxon tests was used for further analysis. The differences in relative gene expression between variants were determined by Welch's t-test. Results with a *p* value less than 0.05 were considered as statistically significant.

## Results

### Construction of an in vitro CAL-29 cell line model

To investigate the relationship between WWOX, AP-2α and AP-2γ in bladder cancer, *WWOX* gene expression was downregulated or upregulated in the CAL-29 cell line. Overexpression of the *WWOX* gene was confirmed at the protein level by Western Blot analysis. A higher level of *WWOX* was observed (*p* = 0.0002) in the WWOX↑ variant (mean protein expression level 4.56 ± 0.50) than in WWOX↑ contr (mean protein expression level 1.12 ± 0.15). Silencing of the *WWOX* gene was also confirmed (*p* = 0.0172) using Western Blot assay (mean protein expression level 0.29 ± 0.08 for sgWWOX vs 1.39 ± 0.19 for sgWWOX contr variants).

The next stage examined whether differences in *WWOX* expression influence AP-2α and AP-2γ level. It was found that level of AP-2γ expression was higher (*p* = 0.0169) in the WWOX↑ variant (mean protein expression level 0.15 ± 0.009) than in WWOX↑ contr (mean protein expression level 0.09 ± 0.007). The same tendency was noticed for AP-2α − expression level was higher (*p* = 0.0002) after *WWOX* gene overexpression (mean protein expression level 0.20 ± 0.017) compared to control (mean protein expression level 0.05 ± 0.01).

**Fig. 1:**
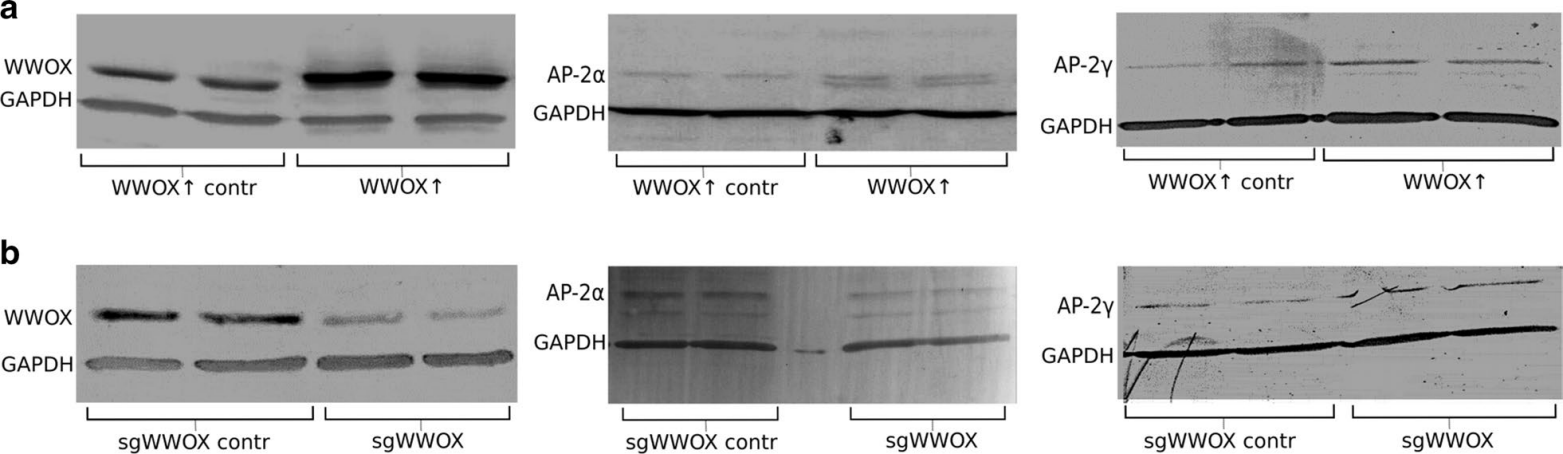
Confirmation of *WWOX* overexpression/downregulation on protein level in CAL-29 cell line with influence on AP-2α/AP-2γ level. **a** *WWOX* overexpression. **b** *WWOX* downregulation

In contrast, *WWOX* silencing resulted in a lower level (*p* = 0.0174) of AP-2α (mean protein expression level 0.08 ± 0.01 vs 0.18 ± 0.01). Surprisingly, the level of AP-2γ remained higher (*p* = 0.0483) in the sgWWOX variant (mean protein expression level 0.14 ± 0.01) than in sgWWOX contr (mean protein expression level 0.05 ± 0.03). The findings are collected in Fig. 1.

All biological experiments were performed on CAL-29 cell line variants with either overexpressed or downregulated *WWOX* gene expression. While the level of AP-2γ was consistently high for all variants, compared to their respective controls, the levels of AP-2α varied.

Numerous significant changes in biological behavior of the investigated cell variants were observed with regard to *WWOX* expression level. These are thoroughly described in the following sections.

### The effect of WWOX on bladder cancer cell proliferation, clonogenic survival and mitochondrial activity

**Fig. 2:**
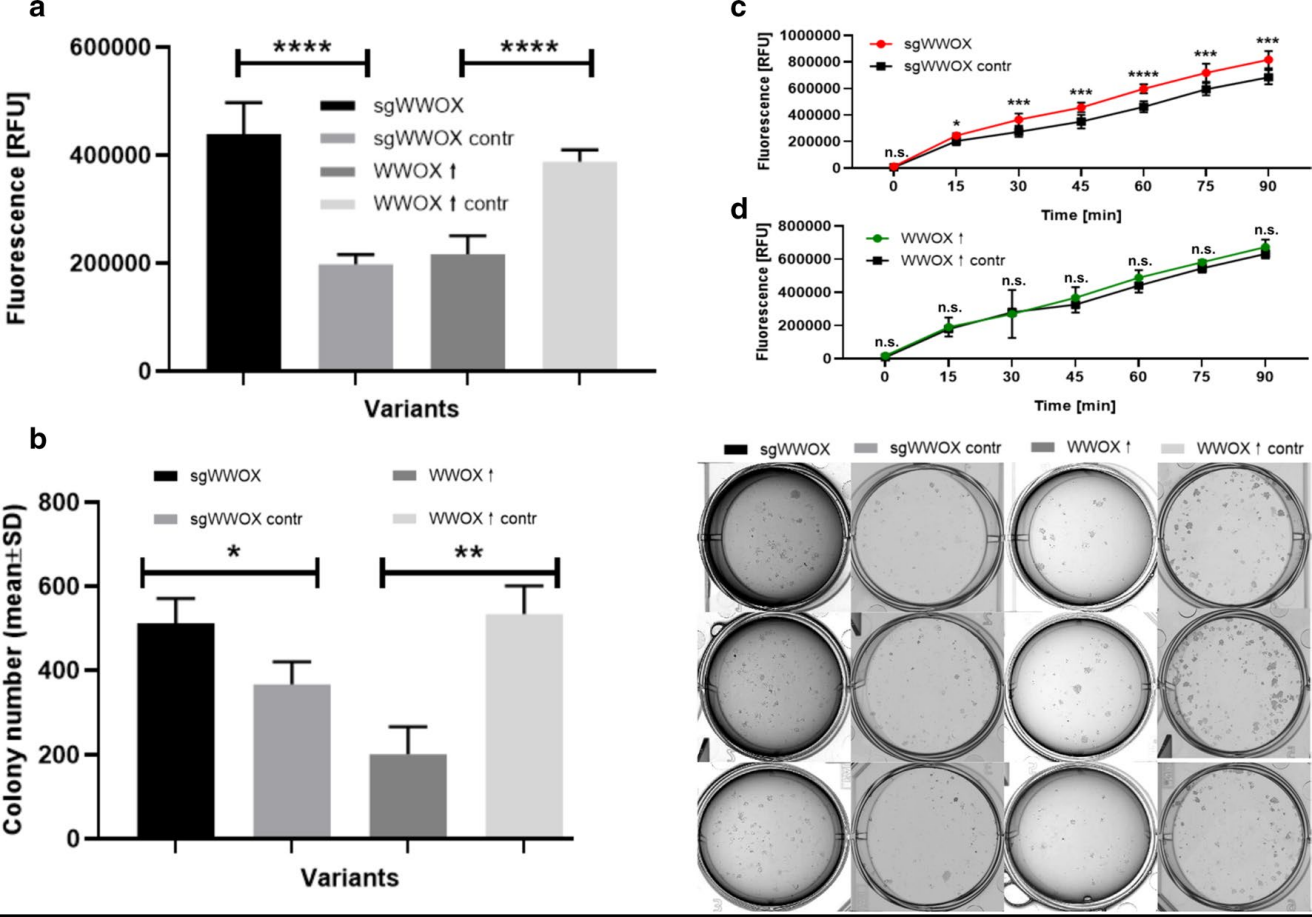
Results of proliferation, colony formation and mitochondrial activity assays for CAL-29 cell line variants. **a** Differences in proliferation potential between CAL-29 variants. *p* < 0.0001 (****). **b** Bar plot presenting number of colonies in all variants of WWOX. *p* < 0.05 (*), *p* < 0.01 (**). The first column of biological assay images shows sgWWOX variant in triplicate. The second row refers to the sgWWOX contr, the third for WWOX↑ and the fourth for WWOX↑ contr. **c** The dependence of mitochondrial activity on WWOX downregulated variants. *p* < 0.05 (*), *p* < 0.001 (***), *p* < 0.0001 (****). **d** The dependence of mitochondrial activity on WWOX overexpressed variants

Reduced proliferation activity was noticed in the WWOX↑ variant (*p* < 0.0001) compared to its control whereas inverse tendency concerned sgWWOX and its reference (Fig. 2a). These observations were consistent with the analysis of the ability of a single cell to grow into a colony. The CAL-29 variant overexpressing WWOX demonstrated a 2.6-fold decrease in the number of colonies compared to WWOX↑ contr (*p* = 0.0036) while variants representing downregulation shown significant (*p* = 0.0342) 1.4-fold opposite trend (Fig. 2b). Lastly, sgWWOX variant was significantly associated with increased mitochondrial redox activity compared to its control (Fig. 2c).

### The influence of WWOX on the invasiveness of bladder cancer cells (metalloproteinases activity, adhesion and migratory potential)

**Fig. 3:**
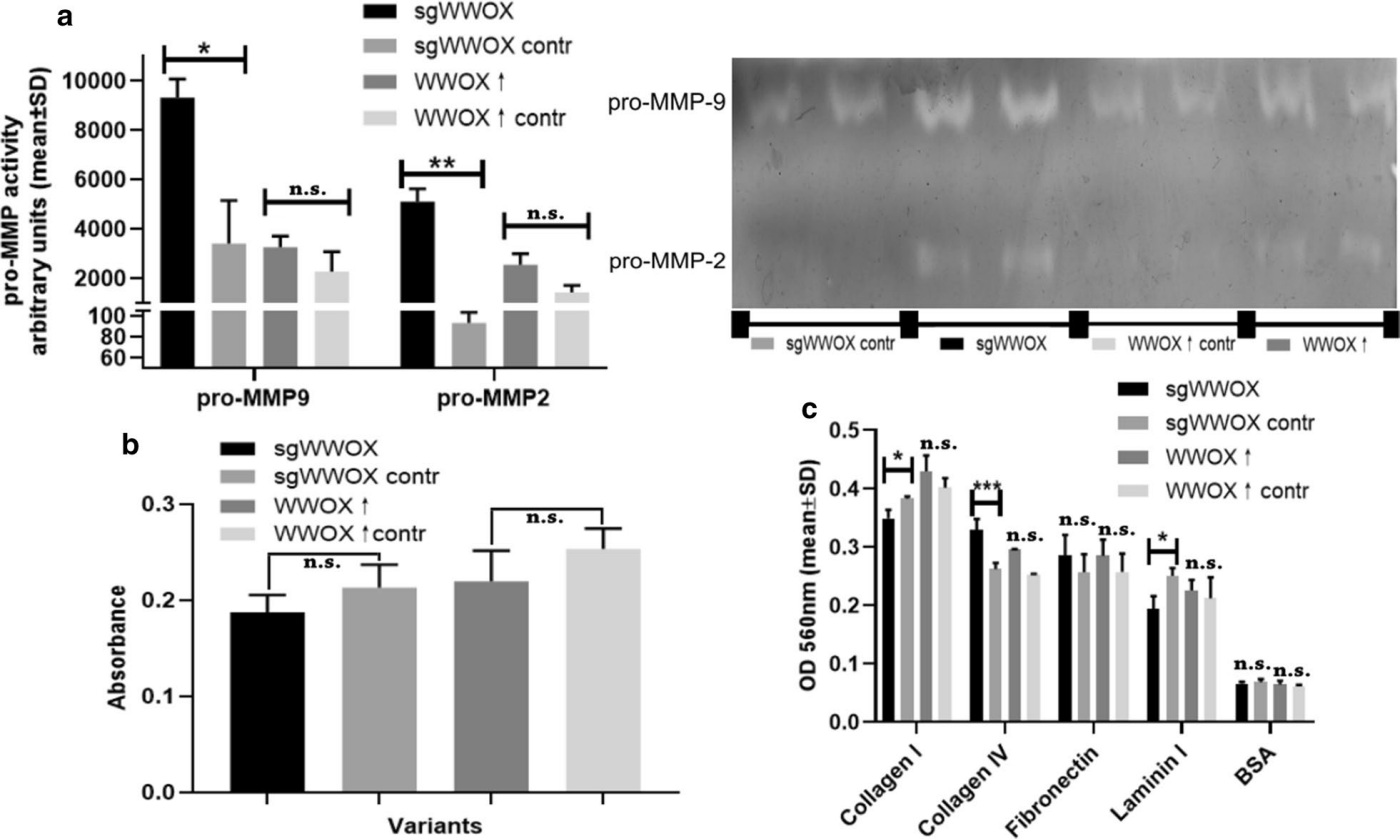
Evaluation of basic biological processes affecting the progression of bladder carcinogenesis. **a** Graph and gelatin gel presenting activity of pro-MMP-2 and pro-MMP-9 in CAL-29 cell line with various level of WWOX gene *p* < 0.05 (*), *p* < 0.01 (**)). **b** Assessment of transmembrane migratory potential based on invasiveness assay. **c** The differences of ability for adhesion to selected extracellular matrix (ECM) proteins of CAL-29 cell line variants. *p* < 0.05 (*), *p* < 0.001 (***)

Downregulation of the *WWOX* gene resulted in more than 10-fold increasement of pro-MMP-2 and 2.7-fold of pro-MMP-9 activities (*p* = 0.0052 and *p* = 0.0481, respectively) (Fig. 3a). Nevertheless, it did not appear to have any significant effect on cell motility through a basal membrane (*p* = 0.0921) (Fig. 3b). Moreover, sgWWOX variant demonstrated a greater ability to adhere to collagen IV (*p* = 0.0008) but not to collagen I, for which it presented inverse character (*p* = 0.0277) (Fig. 3c).

### The influence of WWOX on 3D culture growth

**Fig. 4:**
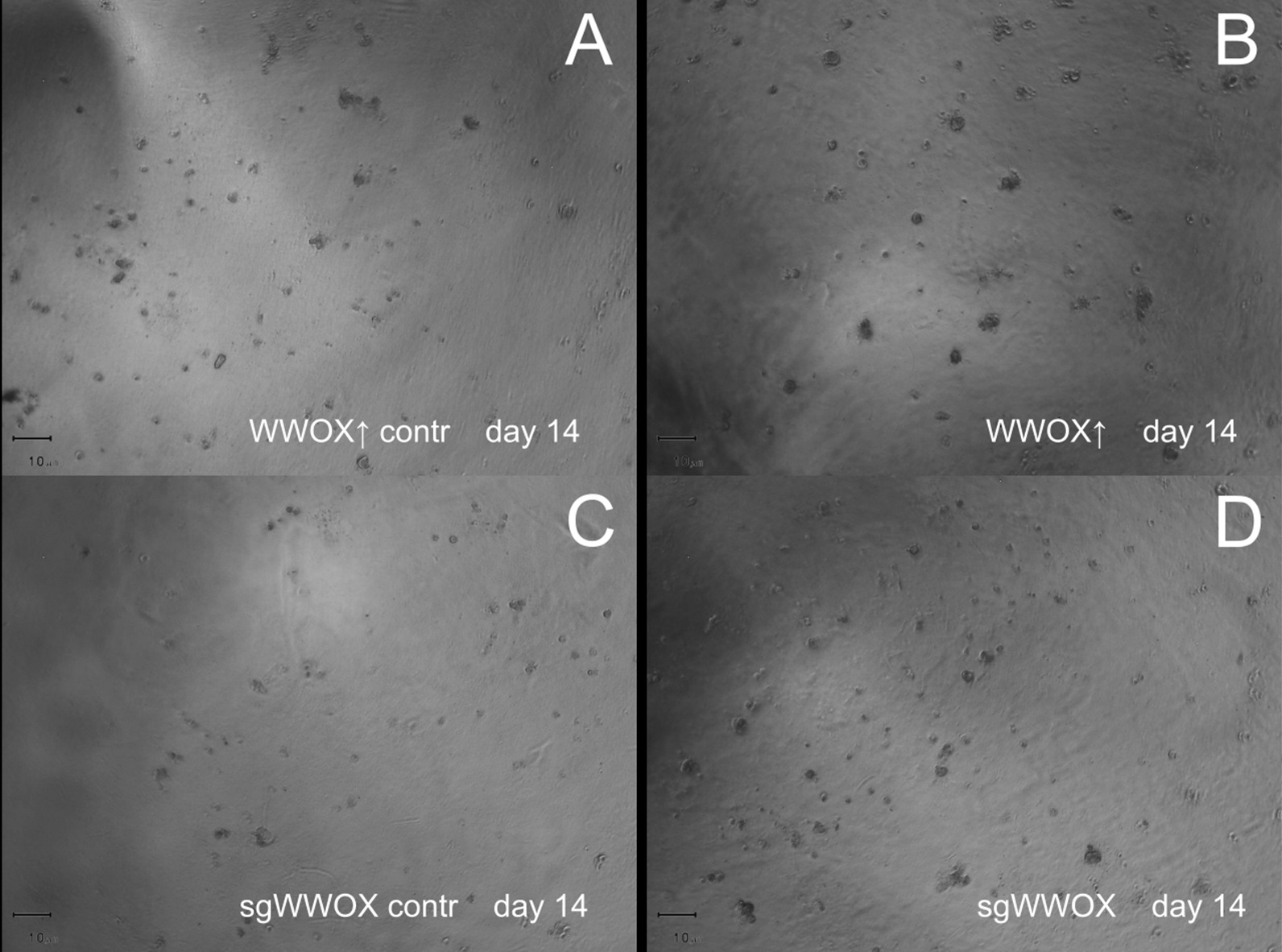
Comparison of the growth of CAL-29 cell line variants in the 3D culture. **a**, **b** WWOX overexpression variants. **c**, **d** WWOX silencing variants

The CAL-29 variants varied according to their ability to organize cells into colonies and form spatial structures, as well as their cell sizes and dissemination in 3D culture growth. The cells with high *WWOX* level eagerly organized themselves into larger aggregates compared to its control (without incrementing the number of separate colonies), while sgWWOX cells not only formed assemblages but they were scattered throughout the substratum on a larger scale than that of sgWWOX contr (Fig. 4).

### Correlation between WWOX level and expression changes of AP-2γ target genes

**Fig. 5:**
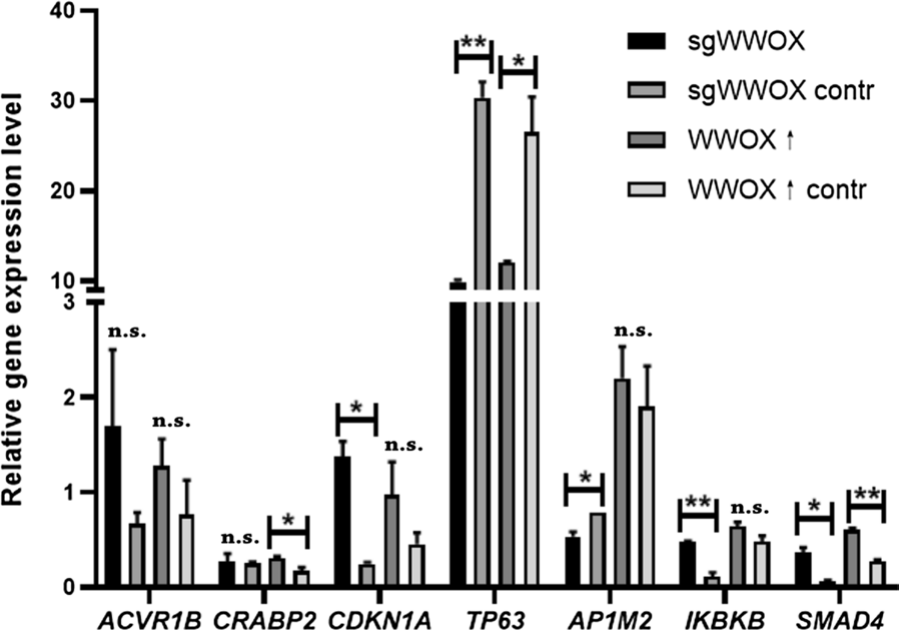
Changes in AP-2γ target genes (mRNA level) depending on WWOX level in CAL-29 cell line. *p* < 0.05 (*), and *p* < 0.01 (**)

The study also aimed to investigate whether WWOX can affect the expression of AP-2γ target genes. In the case of *WWOX* overexpression, the *TP63* or *SMAD4* genes respectively decreased by 2-fold (*p* = 0.0347) or increased by 2-fold (*p* = 0.0037) compared to the appropriate control. Analogously, the same tendencies were observed during *WWOX* silencing (3-fold decrease with *p* = 0.0038 for *TP63*; 5-fold increase with *p* = 0.0183 for *SMAD4*). Furthermore, for variants representing *WWOX* downregulation, in the sgWWOX variant there was a 4-fold increase in *IKBKB* and 5-fold in *CDKN1A* gene expression (*p* = 0.0068 and *p* = 0.0101, respectively) as well as a 1.5-fold decrease in *AP1M2* expression (*p* = 0.0212) compared to sgWWOX contr. Lastly, variants representing *WWOX* overexpression shown a statistically significant (*p* = 0.0487) 1.8-fold increasement in the level of expression for the *CRABP2* gene in the WWOX↑ variant compared to the control (Fig. 5).

### Global profiling of BLCA patients according to *WWOX*, *TFAP2A* and *TFAP2C* expression: in silico analyses

It has been shown that the interplay of *WWOX* and *TFAP2A*/*TFAP2C* affects the biology of the tumor thus corresponding bioinformatics analyses involving BLCA cohort were performed as a follow-up of in vitro experiments.

### *WWOX* modulation of *TFAP2C/TFAP2A* significantly alters disease-free survival of BLCA patients

**Fig. 6:**
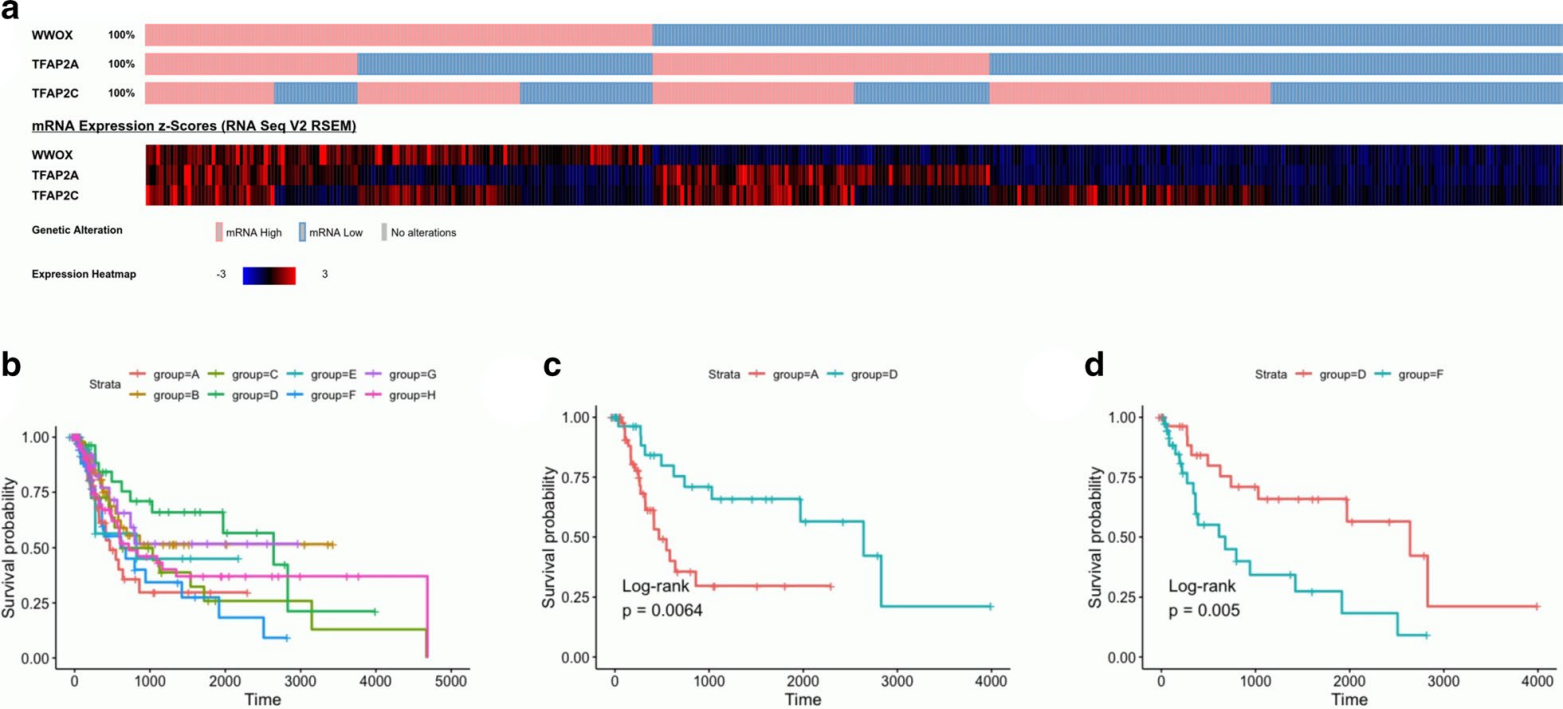
Groups classification based on *WWOX*, *TFAP2A* and *TFAP2C* expression levels. **a** Oncoprint analysis. **b** Survival plot presenting all designated groups. **c** Survival plot comparing group A vs D. **d** Survival plot comparing group D vs F

DFS analysis was applied in groups of patients that were classified according to expression levels of *WWOX*, *TFAP2A* and *TFAP2C* based on oncoprint analysis (Fig. 6a). Eight groups that were considered showed differential survival time (Fig. 6b), among which A vs D (HR = 0.34; Fig. 6c) and D vs F (HR = 2.9, Fig. 6d) were the most relevant indicating essential role of signaling through *TFAP2C* modulated by *WWOX*. More specifically, combination of low *WWOX* and high *TFAP2C*/*TFAP2A* (group A) was less favorable on DFS than high *WWOX* and low *TFAP2C*/*TFAP2A* (group D), whereas high *WWOX* with high *TFAP2C* and low *TFAP2A* (group F) was unfavorable in comparison with high *WWOX* and low both *TFAP2C*/*TFAP2A* (group D). Table 4 presents detailed statistics of DFS analysis.

**Table 4:** Statistical data of disease-free survival analysis

Groups comparison	Hazard Ratio	95% Confidence Interval	*p* value	Log-rank *p* value
A vs D	0.34	0.15–0.76	0.009	0.0064
D vs F	2.9	1.3–6.4	0.007	0.005

### WWOX modulates pivotal biological processes through AP-2γ and AP-2α downstream effectors

**Fig. 7:**
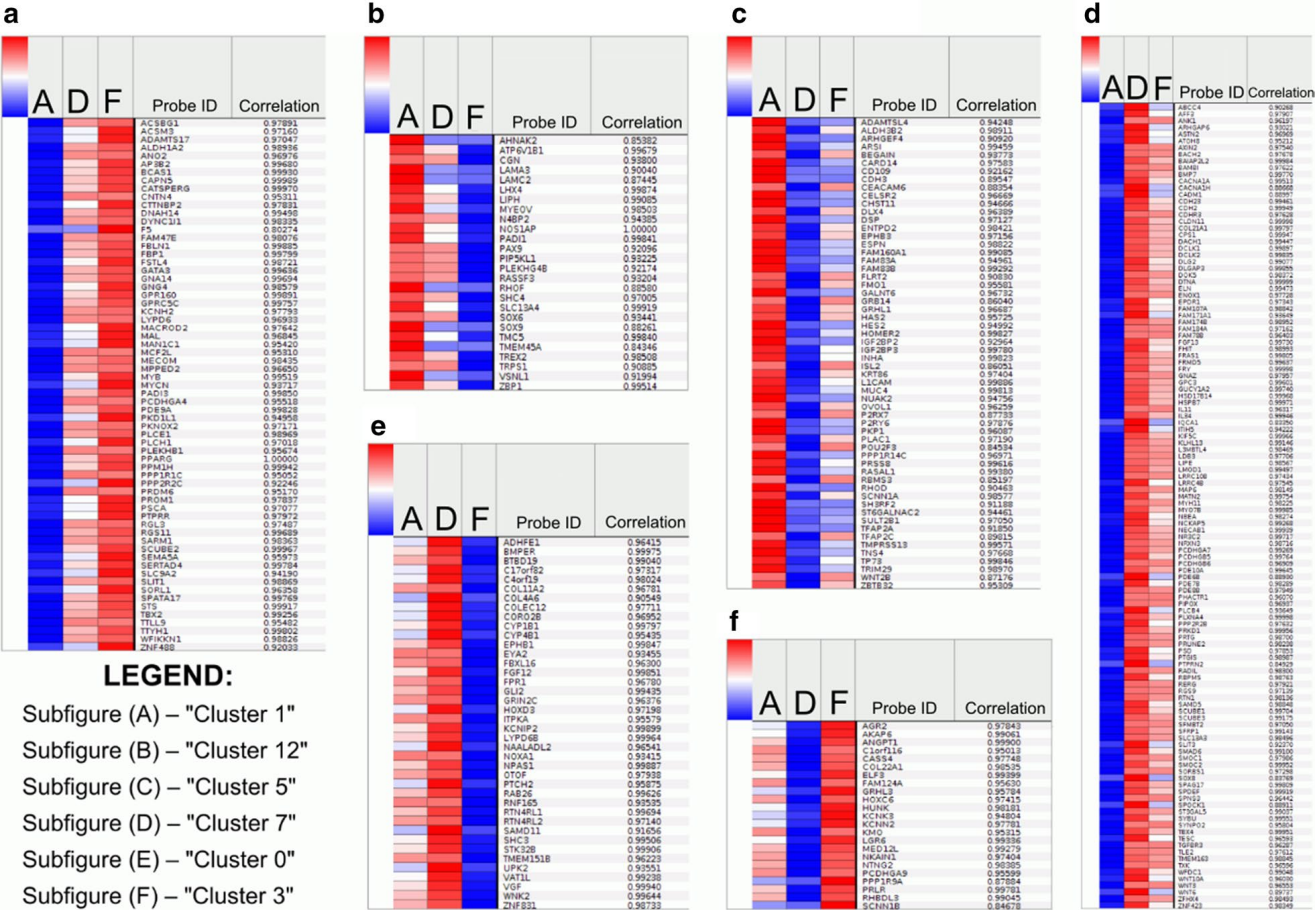
Heatmaps showing expression patterns across most significant AP-2γ targets’ clusters among three most significant groups

Downstream targets of AP-2 factors were clustered among three the most significant groups with respect to DFS analysis (A, D and F) to reveal common and distinct profiles of downstream effectors expression followed by identification of biological processes that altered genes are involved in. Regarding targets of AP-2γ, total of 9 clusters of genes were determined with 3 specific patterns related to DFS groups differing A vs F, A vs D and D vs F groups. Specifically, “Cluster 1” reflected overrepresentation of cell differentiation regulators that were downregulated in A group in contrast to F group, while opposite profile was associated with interferon signaling pathway in “Cluster 12” (Fig. 7a, b). Furthermore, cell to cell adhesion was downregulated in the group D (“Cluster 5”) compared to A group, in contrast to negative regulation of Wnt signaling pathway genes that were downregulated in the latter (“Cluster 7”) (Fig. 7c, d). Finally, cell communication was upregulated in D group and downregulated in F group (“Cluster 0”) in comparison to cell migration presenting opposing expression pattern (“Cluster 3”) (Fig. 7e, f). Remaining expression heatmaps for both transcription factors are included in Additional file 2. Summary of clustering and gene ontology analysis is shown in Table 5.

**Table 5:** Gene ontology summary including AP-2 factors subdivision for the most significant groups from DFS analysis

Transcription factor	Cluster number	Examples of genes	Biological process	Statistical significance (*p* value )
AP-2α	1	*AMOT; SEMA5A*	Regulation of cell migration	0.0258
	2	*PLXNA4; PCDHGB7; DLG2; CDHR3; PCDHGB6; PCDHGB5; PCDHGA7*	Cell adhesion	0.00264
	7	*EYA2*	Positive regulation of DNA repair	0.0222
	11	*NTNG2*	Cell motility	0.0344
	12	*BEGAIN; NTF4; GRB14; WNT2B; HOMER2; INHA*	Cell surface receptor signaling pathway	0.0257
	13	*PDCD1LG2*	Positive regulation of T cell proliferation	0.0161
AP-2γ	0	*COL4A6; STK32B; WNK2*	Cell communication	0.0133
	1	*PLEKHB1; SEMA5A; GATA3*	Regulation of cell differentiation	0.0145
	3	*CASS4*	Cell migration	0.0329
	5	*DSP; PKP1; L1CAM; CELSR2*	Cell–cell adhesion	0.000683
	7	*TLE2; SFRP1*	Negative regulation of canonical Wnt signaling pathway	0.0146
	12	*ZBP1*	Type I interferon signaling pathway	0.00902

## Discussion

The *WWOX* tumor suppressor gene, located in a common fragile site, is involved in the regulation of carcinogenesis in many tissue types [34–38] including bladder cancer [7]. Only a few papers clarified that processes such as promoter methylation or LOH leads to downregulation of *WWOX* expression in BLCA patients [7, 8, 39] and mentioned the connection with a more aggressive cancer phenotype [10]. Therefore, the primary aim of this study was to determine the role of the *WWOX* gene in biological cancer-related processes in the aggressive bladder cancer cell line CAL-29 (derived from grade 4 tumor) and identify its connections with the AP-2 family of transcription factors. Our in silico analysis indicated various pathways that might be modulated by changes in the expression of the *WWOX* and *TFAP2C* genes.

Several biological assays were performed to investigate the specific effects of *WWOX* on tumor cell behavior regarding invasiveness in CAL-29 cell line. Firstly, a cell variant with high *WWOX* expression was found to demonstrate 2.6-fold reduction of anchorage-independent clonogenicity. Similar results have been noticed during in vitro experiments on other cancer types such as breast cancer [11], endometrial adenocarcinoma [34, 40], the endometrial cancer cell line ECC1 [41], non-small cell lung cancer [42] or colon cancer [43]. Such trend is further supported by comparison of downregulated WWOX to its control, where sgWWOX variant exhibited higher clonogenicity than sgWWOX contr.

Moreover, the CAL-29 cell line with WWOX downregulation demonstrated disparate adhesion to collagen I, laminin and collagen IV with the first two decreased while the latter increased. Same observation of cells attachment to collagen I and laminin after *WWOX* silencing has been noticed in vitro for the THESC cell line [34] and additionally for laminin in MCF10 cell line [24]. This can be further supported by confirmation that collagen I expression in colon cancer HT29 cells significantly increases during *WWOX* overexpression [43], indicating our results for sgWWOX variant to form logical whole by complementing this statement. However, the results for collagen IV are inverse compared to the available literature [24], but we consider that these differences may reflect various processes of cell remodeling which depends on tissue type or cancer differentiation grade.

Our results also indicated that changes in *WWOX* expression influence the spatial growth of tumor cells. Meaning, cells having high *WWOX* level exhibited bigger colonies however no quantitative increase has been noted compared to control. Contrastingly, cells with WWOX silencing induced both colony size and formation of newly separated cell habitats. This is consistent with the effect of *WWOX* on the 3D growth of T98G glioblastoma cells for which the amount of emerging colonies along with the proliferation rate were examined [44]. Likewise, a promotion in the activity of the pro-MMP-2 and pro-MMP-9 metalloproteinase enzymes was noted in the sgWWOX variant, which may suggest an enlarged ECM-degrading potential [45].

The WWOX↑ variant also demonstrated 43% lower proliferation potential than control, which together with confirmatory trend between sgWWOX vs sgWWOX contr, is in line with results obtained for osteosarcoma and glioblastoma [44, 46]. Additionally, those changes in AP-2γ targets expression that can be considered simultaneously between sgWWOX and WWOX↑ vs. controls (i.e. only *TP63* and *SMAD4*) suggest no effect of WWOX on expression of certain AP-2γ targets. However, this is not necessarily the case with all genes, as exemplified by the tendencies visible for *CDKN1A* and *IKBKB*, which have references in the literature. In the former, an expression increase of the gene encoding the p21 protein was observed during loss of WWOX expression in breast cancer [47]. For the latter, the only report concerns primary effusion lymphomas (PELs) in which the loss of suppressor genes such as *WWOX*, fragile histidine triad (*FHIT*) or glutamate receptor ionotrophic delta 2 (*GRID2*) is observed. In one of the PEL subsets, *IKBKB* amplification is observed among others, which is not a direct confirmation of the relationship between *WWOX* and *IKBKB* expression, but gives a hint about their potential relationship [48].

Ultimately, CAL-29 cells representing *WWOX* downregulation demonstrated a significant increase in cell viability. This proves that potentiated mitochondrial activity is visible during *WWOX* depletion, which is consistent with available literature [49]. Additionally, overexpression of *WWOX* led to a decrease in cell viability of colon cancer cell line SW480 which complement our observations for silenced *WWOX* variant [43]. The aforementioned results confirmed that *WWOX* can regulate a wide range of biological processes taking place in the advanced stage of bladder cancer. As the WWOX interacts with many proteins including transcription factors AP-2 α and γ [26], the present study examined relationship between these proteins in bladder cancer hence we also pursued this issue in further bioinformatics analyzes.

A correlation analysis of disease-free survival time with *WWOX*, *TFAP2C* and *TFAP2A* levels revealed that in BLCA patients the most favorable is high *WWOX* expression with simultaneously low *TFAP2C* and *TFAP2A* (Fig. 6b, group D). Our results from the experimental section on CAL-29 cell line are consistent with in silico analysis for the *WWOX* overexpressing model. Furthermore, we obtained statistically significant results for groups characterized by low *WWOX* and high *TFAP2C*/*TFAP2A* expression (group A), but also high *WWOX*/*TFAP2C* and low *TFAP2A* (group F), in which modifications of *TFAP2C* and *TFAP2A* levels worsen DFS in comparison to the group D. Thus, we decided to perform independent clustering of AP-2γ or AP-2α targets to investigate differentially expressed genes among groups A, D, F with subsequent examination of processes in which AP-2 downstream effectors are implicated in. Considering groups of AP-2γ targets, two Clusters (“0” and “3”) seemed to explain the nature of this transcription factor most accurately since these are the only clusters in which two groups differing only in *TFAP2C* expression (D and F, respectively *TFAP2C* low and high) have inverse pattern of gene expression.

By profound analyzing both Fig. 7 and Table 5, the main noticeable trend between AP-2γ targets in Clusters “0” and “3” and their expression in groups D and F is that the level of AP-2γ negatively correlates with the expression of their targets from “Cluster 0”, while it correlates positively with its effectors from “Cluster 3”. In “Cluster 0” we observed genes upregulated in group D which further supported its favorable outcome during survival analysis. Examples are *COL4A6*, *WNK2* and *STK32B* whose roles have been assigned in processes such as prevention of early invasion stages, negative regulation of epidermal growth factor receptor signaling and opposite correlation with tumor size, respectively [50–52]. Regarding "Cluster 3" which was elevated in group F, the gene capable to represent the tendency is *CASS4* whose overexpression has been associated with promotion of lung cancer invasion by inhibiting E-cadherin expression and activating AKT signaling pathway [53]. This is consistent with gene ontology description indicated by the bioinformatics database, which concerns cell migration for “Cluster 3”.

Nevertheless, we consider it appropriate to broaden the analysis of the remaining clusters not only obtained from the AP-2γ targets but also from AP-2α analysis, where processes such as cell motility, migration, positive regulation of T-cell proliferation, cell adhesion or DNA repair were noted. However, due to the lack of proper and significant bioinformatics model that will directly explain *TFAP2A* participation in specific biological processes, we cannot conclude about its characteristics. Following in vitro assays are required to elaborate the properties of both AP-2γ and AP-2α factors depending on different levels of the *WWOX* gene.

## Conclusions

To conclude, our findings suggest that *WWOX* deficiency may have an impact on the invasion process in the bladder. This is supported by the fact that during *WWOX* downregulation, an increase in both mitochondrial redox potential (indicating greater cell viability) or the metalloproteinases’ activity (suggesting ECM-degrading characteristics) were observed for sgWWOX CAL-29 cell line variant. Moreover, these cells demonstrated higher proliferation potential and ability of a single cell to grow into a colony. The subsequent analyzes are required to reveal the additional contribution of AP-2γ or AP-2α.

Bioinformatics analyses in bladder cancer patients found high expression of *TFAP2C* gene to be associated with an enhancement of adverse processes related to cancer progression, probably due to its oncogenic nature. Further research based on the dual modification of the AP-2α and AP-2γ expression is needed to precisely determine the nature of the interaction between *WWOX* and particular transcription factors in bladder cell line models also in different stage of cell differentiation.

## Supplementary Information

**Additional file 1**. Collective characteristics of BLCA patients

**Additional file 2.** Heatmaps showing expression patterns across clusters of AP-2α and AP-2γ targets

## Data Availability

The datasets generated and/or analysed during the current study are available in the Broad GDAC Firehose repository, http://firebrowse.org/?cohort=BLCA.

## Abbreviations

WWOX: WW Domain Containing Oxidoreductase
AP-2: Activator Protein 2
BLCA: Bladder cancer
UNC: University of North Carolina
MDA: MD Anderson
TCGA: The Cancer Genome Atlas
LOH: Loss of heterozygosity
CSE: Cigarette smoke extract
TSS: Transcription start site
SDR: Short chain dehydrogenase domain
ECM: Extracellular matrix
DSMZ: Deutsche Sammlung von Mikroorganismen und Zellkulturen
FBS: Fetal Bovine Serum
PSN: Penicillin; Streptomycin; Neomycin
DFS: Disease-free survival
HR: Hazard ratio
GTRD: Gene Transcription Regulation Database
TRANSFAC: TRANScription FACtor database
TRRUST: Transcriptional Regulatory Relationships Unraveled by Sentence-based Text mining
PANTHER: Protein ANalysis THrough Evolutionary Relationships
PMSF: Phenylmethylsulfonyl fluoride
REST-MCS: Relative Expression Software Tool Multiple Condition Solver
BrdU: 5-Bromo-2′-deoxyuridine
PELs: Primary effusion lymphomas
FHIT: Fragile histidine triad
GRID2: Glutamate receptor ionotrophic delta 2
