# Supplementary material for: In vitro and in silico assessment of the effect of WWOX expression on invasiveness pathways associated with AP-2 transcription factors in bladder cancer
Source: BMC Urol. 2021 Mar 10;21:36. doi: 10.1186/s12894-021-00806-7 (PMC7944886; doi:10.1186/s12894-021-00806-7)
Supplement: Supplementary file 1 — Additional file 1. Collective characteristics of BLCA patients [file 12894_2021_806_MOESM1_ESM.docx]

**Additional file 1.** Collective characteristics of BLCA patients.

| group | A | B | C | D | E | F | G | H | all |
| --- | --- | --- | --- | --- | --- | --- | --- | --- | --- |
| General characteristics of patients | | | | | | | | | |
| median age [in years] (range) | 66.5 (34-86) | 69 (41-89) | 67 (37-83) | 67.5 (44-86) | 67 (54-85) | 72 (47-90) | 66 (47-90) | 71 (48-88) | 69 (34-90) |
| Gender:  Male  Female | 41  17 | 60  24 | 30  7 | 27  11 | 18  6 | 31  16 | 35  4 | 59  22 | 301  107 |
| Race:  Asian  Black or African American  White  NAs | 4  6  47  1 | 15  3  64  2 | 3  3  29  2 | 9  3  26  - | 1  -  20  3 | 2  3  38  4 | 5  2  30  2 | 5  3  70  3 | 44  23  324  17 |
| median height [cm] (range) | 175 (152.4-187) | 172 (149-194) | 174.8 (152-193) | 168 (152-185) | 173 (152-195.6) | 175 (139-196) | 172 (152-188) | 173 (145-190) | 172.7 (139-196) |
| median weight [kg] (range) | 83 (48-142.88) | 75 (41-148) | 80 (54-128) | 71 (52-93) | 85 (58-121) | 80 (48-150) | 75 (45-133) | 77 (44-292) | 77 (41-292) |
| median BMI (range) | 27.76 (17.63-49.33) | 25.71 (14.96-46.38) | 25.68 (19.69-36.59) | 25.04 (17.99-31.44) | 26.72 (20.88-36.75) | 26.64 (17.01-68.32) | 24.57 (17.58-41.51) | 26.72 (18.82-48.73) | 25.98 (14.96-68.32) |
| Smoking-associated characteristics of patients | | | | | | | | | |
| median age began smoking [in years] (range) | 18 (10-57) | 17 (9-53) | 20 (13-23) | 22 (15-42) | 18 (14-38) | 19 (12-44) | 20 (11-51) | 20.5 (9-50) | 20 (9-57) |
| Smoking history:  1^[[1]](#footnote-1)^  2^[[2]](#footnote-2)^  3^[[3]](#footnote-3)^  4^[[4]](#footnote-4)^  5^[[5]](#footnote-5)^  NAs | 15  13  20  7  2  1 | 21  23  21  11  4  4 | 8  8  15  4  1  1 | 13  9  3  6  -  2 | 3  4  13  3  1  - | 10  8  16  10  2  1 | 16  10  3  7  1  2 | 23  14  17  24  1  2 | 109  89  113  72  12  13 |
| Clinical characteristics of patients | | | | | | | | | |
| Cancer status:  With tumor  Tumor free  NAs | 21  30  7 | 19  61  4 | 19  17  1 | 11  22  5 | 7  15  2 | 19  20  8 | 9  26  4 | 30  43  8 | 135  234  39 |
| Vital status:  Dead  Alive | 18  40 | 23  61 | 12  25 | 9  29 | 7  17 | 12  35 | 7  32 | 20  61 | 108  300 |
| Pathologic stage:  I  II  III  IV  NAs | -  13  22  23  - | -  41  25  18  - | -  11  13  13  - | 1  13  16  8  - | -  6  12  6  - | -  13  13  21  - | -  15  15  8  1 | 1  18  24  37  1 | 2  130  140  134  2 |
| Histologic grade:  High  Low  NAs | 57  1  - | 76  8  - | 35  2  - | 35  3  - | 22  2  - | 46  -  1 | 35  4  - | 78  1  2 | 384  21  3 |
| Gleason score:  5  6  7  8  9  NAs | -  7  4  -  -  47 | 1  9  4  -  -  70 | -  5  3  -  1  28 | -  4  2  -  -  32 | -  4  2  -  -  18 | 1  5  4  1  -  36 | -  8  2  -  -  29 | -  11  6  -  -  64 | 2  53  27  1  1  324 |
| Disease-specific characteristics of patients | | | | | | | | | |
| Anatomic neoplasm subdivision:  Bladder - nos  Dome  Neck  Trigone  Wall anterior  Wall lateral  Wall nos  Wall posterior  NAs | 30  2  1  5  3  6  4  7  - | 41  3  2  8  3  13  8  6  - | 24  1  -  2  5  1  1  2  1 | 21  3  -  3  1  4  2  4  - | 10  3  -  2  -  3  1  3  2 | 20  4  1  1  3  5  2  9  2 | 25  1  -  3  1  6  2  1  - | 35  7  4  7  10  8  2  8  - | 206  24  8  31  26  46  22  40  5 |
| Diagnosis subtype:  Non-papillary  Papillary  NAs | 45  12  1 | 47  37  - | 27  10  - | 23  15  - | 14  8  2 | 32  14  1 | 26  13  - | 57  23  1 | 271  132  5 |
| Histological type:  Muscle invasive urothelial carcinoma (pt2>)  NAs | 58  - | 83  1 | 37  - | 37  1 | 24  - | 47  - | 39  - | 80  1 | 405  3 |
| Lymphovascular invasion present:  Yes  No  NAs | 24  18  16 | 25  23  36 | 11  14  12 | 11  16  11 | 9  7  8 | 22  15  10 | 11  17  11 | 38  20  23 | 151  130  127 |
| Primary lymph node presentation assessment:  Yes  No  NAs | 42  9  7 | 53  25  6 | 29  5  3 | 25  12  1 | 20  3  1 | 37  3  7 | 26  9  4 | 60  13  8 | 292  79  37 |
| Summary of treatment | | | | | | | | | |
| Postoperative RX^[[6]](#footnote-6)^ TX^[[7]](#footnote-7)^:  Yes  No  NAs | 13  26  19 | 13  44  27 | 9  16  12 | 8  19  11 | 3  9  12 | 5  18  24 | 6  17  16 | 20  35  26 | 77  184  147 |
| Radiation therapy:  Yes  No  NAs | 3  37  18 | 3  58  23 | 2  23  12 | 1  28  9 | -  13  11 | 1  25  21 | -  25  14 | -  56  25 | 10  265  133 |
| Therapy outcome success:  Complete remission/response  Partial remission/response  Progressive disease  Stable disease  NAs | 13  1  12  5  27 | 37  2  10  2  33 | 17  2  2  2  14 | 17  1  3  3  14 | 9  1  1  -  13 | 12  3  4  4  24 | 22  -  3  1  13 | 25  6  7  5  38 | 152  16  42  22  176 |

1. Lifelong non-smoker (<100 cigarettes smoked in lifetime) [↑](#footnote-ref-1)
2. Current smoker (includes daily and nondaily smokers) [↑](#footnote-ref-2)
3. Current reformed smoker for > 15 years [↑](#footnote-ref-3)
4. Current reformed smoker for <= 15 years [↑](#footnote-ref-4)
5. Current reformed smoker (duration not specified) [↑](#footnote-ref-5)
6. Prescription; any medication or treatment ordered [↑](#footnote-ref-6)
7. Treatment [↑](#footnote-ref-7)
