# Supplementary figures and images for: In vitro and in silico assessment of the effect of WWOX expression on invasiveness pathways associated with AP-2 transcription factors in bladder cancer
Source: BMC Urol. 2021 Mar 10;21:36. doi: 10.1186/s12894-021-00806-7 (PMC7944886; doi:10.1186/s12894-021-00806-7)

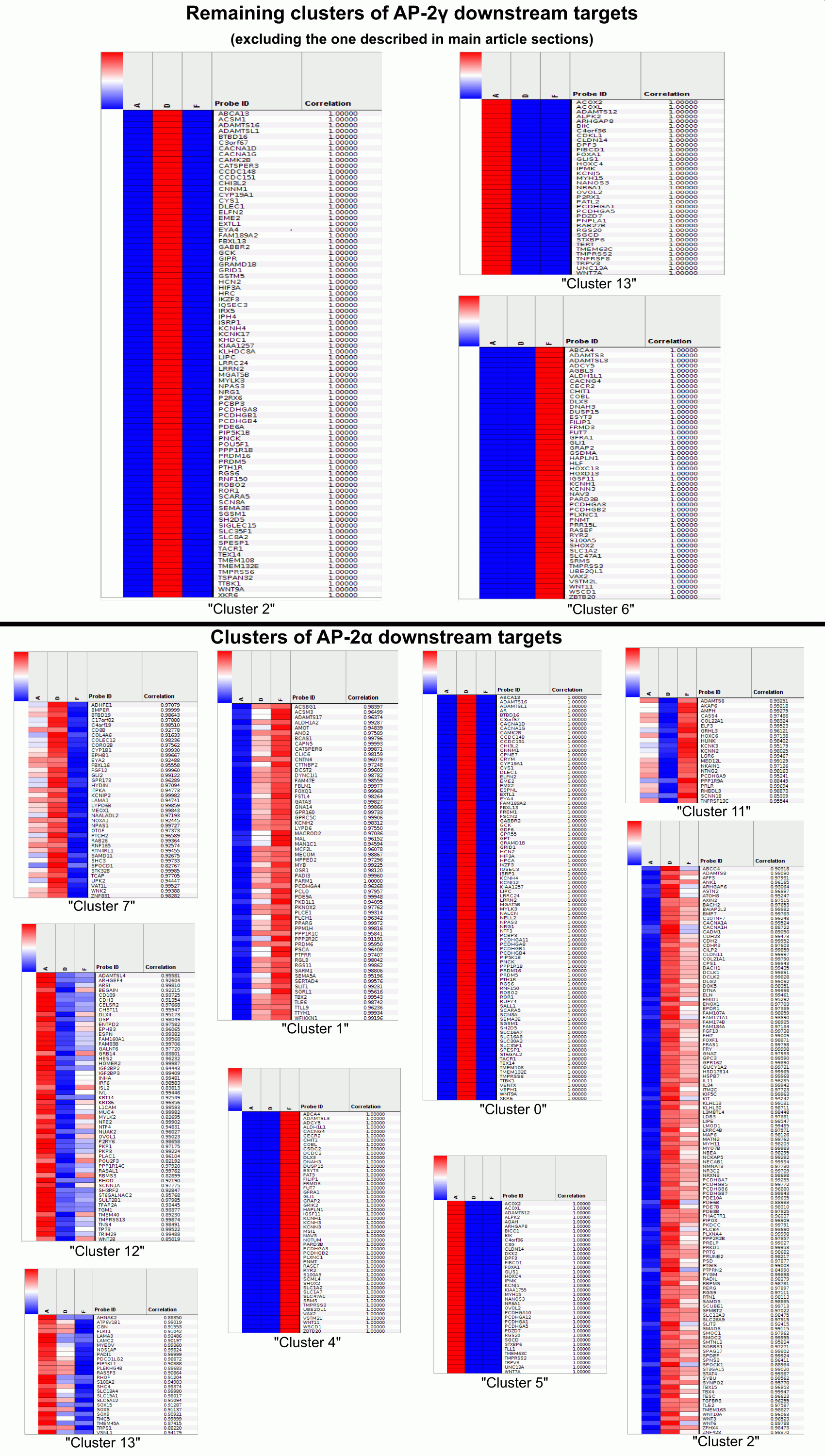

Supplement: Supplementary file 2 — Additional file 2. Heatmaps showing expression patterns across clusters of AP-2α and AP-2γ targets [file 12894_2021_806_MOESM2_ESM.tif]
